# Beyond the Gut: Unveiling Butyrate’s Global Health Impact Through Gut Health and Dysbiosis-Related Conditions: A Narrative Review

**DOI:** 10.3390/nu17081305

**Published:** 2025-04-09

**Authors:** Arda Erkan Kalkan, Mona N. BinMowyna, António Raposo, Md Faruque Ahmad, Faiyaz Ahmed, Abdullah Y. Otayf, Conrado Carrascosa, Ariana Saraiva, Sercan Karav

**Affiliations:** 1 Department of Molecular Biology and Genetics, Çanakkale Onsekiz Mart University, Çanakkale 17100, Turkey; ardaerkankalkan@gmail.com; 2 College of Education, Shaqra University, Shaqra 11911, Saudi Arabia; m.mwena@su.edu.sa; 3 CBIOS (Research Center for Biosciences and Health Technologies), Universidade Lusófona de Humanidades e Tecnologias, Campo Grande 376, 1749-024 Lisboa, Portugal; 4 Department of Clinical Nutrition, College of Nursing and Health Sciences, Jazan University, Jazan 45142, Saudi Arabia; mfahmd@gmail.com (M.F.A.); ayateef@jazanu.edu.sa (A.Y.O.); 5 Department of Basic Health Sciences, College of Applied Medical Sciences, Qassim University, P.O. Box 6666, Buraydah 51452, Saudi Arabia; f.masfoor@qu.edu.sa; 6 Department of Animal Pathology and Production, Bromatology and Food Technology, Faculty of Veterinary, Universidad de Las Palmas de Gran Canaria, Trasmontaña s/n, 35413 Arucas, Spain; conrado.carrascosa@ulpgc.es; 7 Research in Veterinary Medicine (I-MVET), Faculty of Veterinary Medicine, Lisbon University Centre, Lusófona University, Campo Grande 376, 1749-024 Lisboa, Portugal; ariana.saraiva@ulusofona.pt; 8 Veterinary and Animal Research Centre (CECAV), Faculty of Veterinary Medicine, Lisbon University Centre, Lusófona University, Campo Grande 376, 1749-024 Lisboa, Portugal

**Keywords:** butyrate, histone deacetylase inhibitor, neurological protector, obesity, short-chain fatty acids, type 2 diabetes

## Abstract

Short-chain fatty acids (SCFAs), mainly produced by gut microbiota through the fermentation process of dietary fibers and proteins, are crucial to human health, with butyrate, a famous four-carbon SCFA, standing out for its inevitably regulatory impact on both gut and immune functions. Within this narrative review, the vital physiological functions of SCFAs were examined, with emphasis on butyrate’s role as an energy source for colonocytes and its ability to enhance the gut barrier while exhibiting anti-inflammatory effects. Knowledge of butyrate synthesis, primarily generated by Firmicutes bacteria, can be influenced by diets with specifically high contents of resistant starches and fiber. Butyrate can inhibit histone deacetylase, modulate gene expression, influence immune functionality, and regulate tight junction integrity, supporting the idea of its role in gut barrier preservation. Butyrate possesses systemic anti-inflammatory properties, particularly, its capacity to reduce pro-inflammatory cytokines and maintain immune homeostasis, highlighting its therapeutic potential in managing dysbiosis and inflammatory diseases. Although butyrate absorption into circulation is typically minimal, its broader health implications are substantial, especially regarding obesity and type 2 diabetes through its influence on metabolic regulation and inflammation. Furthermore, this narrative review thoroughly examines butyrate’s growing recognition as a modulator of neurological health via its interaction with the gut–brain axis. Additionally, butyrate’s neuroprotective effects are mediated through activation of specific G-protein-coupled receptors, such as FFAR3 and GPR109a, and inhibition of histone deacetylases (HDACs). Research indicates that butyrate can alleviate neurological disorders, including Alzheimer’s, Parkinson’s, autism spectrum disorder, and Huntington’s disease, by reducing neuroinflammation, enhancing neurotransmitter modulation, and improving histone acetylation. This focus will help unlock its full therapeutic potential for metabolic and neurological health, rather than exclusively on its well-known benefits for gut health, as these are often interconnected.

## 1. Introduction

### 1.1. Introduction to Short-Chain Fatty Acids

The gut microbiota, comprising a diverse array of molecules and metabolites, is known to exert a significant impact on host health. Short-chain fatty acids (SCFAs), mainly composed of acetate, propionate, and butyrate, are organic acids synthesized within the gut through bacterial fermentation processes [1]. This fermentation mainly occurs with carbohydrates such as resistant starch and dietary fiber, as well as dietary proteins [2]. SCFAs, acknowledged as intermediaries between host physiology and the microbiota, are non-digestible carbohydrates found in the human colon, with total concentrations reaching up to a maximum of 200 mM [3]. It is believed that SCFAs may be responsible for up to 70% of the energy needs of large intestine cells and even up to 15% of the energy needs for routine human metabolism [4]. Another source indicates that the human intestine generates approximately 400 to 600 mmol of SCFAs daily, with only a minor fraction (~10 mmol) being excreted in feces. The majority of SCFAs are swiftly absorbed by the host’s epithelial cells through passive diffusion or active transport mechanisms [5,6]. Therefore, a dramatic fall in SCFA levels can result in metabolism-oriented diseases [7]. A significant investigation revealed a novel mechanism pathway through which SCFAs inhibit inflammation [8]. Prebiotics play a crucial role in stimulating the production of SCFAs by probiotics. One study demonstrated that the physical state of prebiotic substrates significantly influenced the rate of fermentation and the production of SCFAs [9]. In addition, the concentration of prebiotic levels was shown to have a substantial impact on the quantity of SCFAs produced by probiotics. While Fehlbaum and their team focused on five different prebiotics, which are consecutively inulin, α-galactooligosaccharides (α-GOS), β-galactooligosaccharides (β-GOS), xylo-oligosaccharides, and β-glucan, these prebiotics were carefully studied to determine their specific effects on gut microorganisms at varying concentrations [10]. Last but not least, another discovery was pointed out by Fei et al. [11]. Prebiotics were seen as an agent capable of being fermented into SCFAs, and this ability could offer many advantages for probiotics. Considering these benefits, it was also found that probiotics uniquely gained more opportunities due to the reinforcement of tight junctions. Even further, the proliferation of colon cells was stimulated, and the production of mucus was enhanced, while the total pH average of the intestine was significantly reduced. On top of that, these SCFAs, which went through the production process and were gained from it, served as primary nutrients and were highly effective for probiotic functions in specific ways [12]. Therefore, it is strongly evident that butyrate has an influential role in the gut environment and, on top of that, in expected gut health maintenance. However, butyrate’s roles are not limited to these traits but also involve other and/or related pathways that affect different parts of the body and metabolism. In this paper, butyrate’s most remarkable effects against dysbiosis and the maintenance of the gut health, along with its underestimated other beneficial effects, are thoroughly discussed.

### 1.2. Introduction to Butyrate

As discussed, SCFAs play crucial and vastly important roles throughout metabolism, with butyrate emerging as one of the most studied and frequently mentioned SCFAs within its class, as shown later in this paragraph.

Butyrate plays a crucial role in the regulation of gut microbiota metabolism [13]. Additionally, butyrate works primarily as a metabolic substrate for colonocytes, with up to 90% of it being utilized by these cells [14]. Therefore, butyrate definitely affects these cells’ growth status [15]. In addition, it has been observed that butyrate not only acts as a key regulator in metabolism but also regulates homeostasis in immunological and inflammatory response [14]. In summary, butyrate is known to exert numerous advantageous effects on the human body. Butyrate is prominently abundant in milk and milk products derived from various mammals, as these consumables contain high levels of various types of SCFAs [16]. Significant sources of butyric acid include bovine milk fat and its derivatives, with butter containing approximately 3 g per 100 g, parmesan cheese containing approximately 1.5 g per 100 g, goat’s cheese containing approximately 1–1.8 g per 100 g, and whole cow’s milk containing approximately 0.1 g per 100 g [17]. Butyrate production contributes efficiently to dietary fiber foods, as SCFAs are all abundant and since butyrate is part of SCFAs, such as oats, chickpeas, broccoli, and carrots, since they can be fermented in the gut [18,19]. Meanwhile, foods rich in resistant starches, such as cooked potatoes and green bananas, can contribute to butyrate metabolism [20]. Several certain substrates have been shown to specifically enhance the production of butyrate, such as arabinoxylan-type polysaccharides. These polysaccharides are commonly found in cereal grains and bran, including those derived from wheat, rye, and oats [21,22].

**Figure 1:**
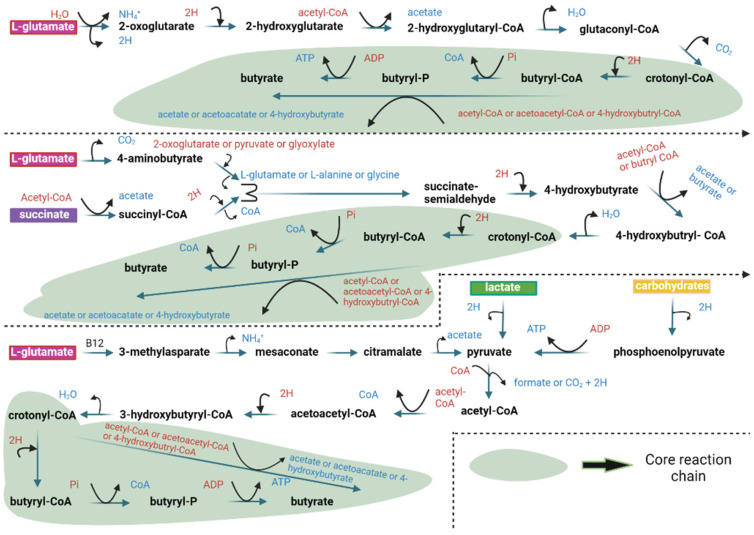
Microbial pathways for butyrate formation from carbohydrates, organic acids, and glutamate in gut communities [3].

**Figure 2:**
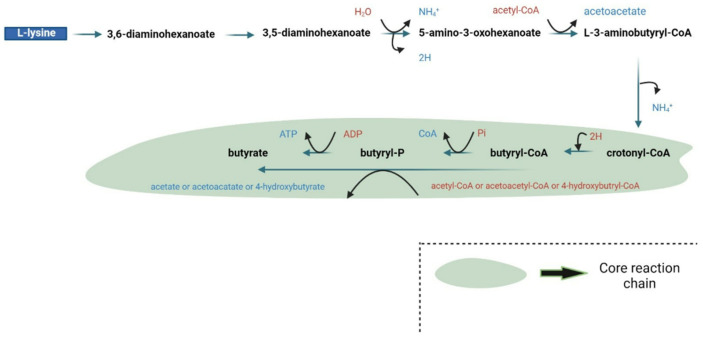
Microbial pathways for butyrate formation from lysine in gut communities [3].

Butyrate also acts as an inhibitor against a class of enzymes named histone deacetylases [23]. Histone deacetylases (HDACs) act by removing the ε-N-acetyl groups from lysine residues leading to the formation of a condensed and transcriptionally silent chromatin by allowing the histones to wrap the DNA more tightly [24,25]. Among the SCFAs, butyrate is the most potent in inhibiting HDAC activities both in vitro and in vivo [26]. Butyrate can be metabolized through two pathways: The first involves butyryl-CoA/acetate CoA transferase, while the second utilizes phosphotransbutyrylase-butyrate kinase [27]. Additionally, in the first pathway, butyrate is produced through glycolysis of carbohydrates, leading to the formation of acetoacetyl-CoA by combining two molecules of acetyl-CoA, which is then reduced to form butyryl-CoA [27]. Microbial pathways for butyrate formation from carbohydrates, organic acids, and glutamate are depicted in Figure 1, while glutamate and lysine in gut communities are illustrated in Figure 2. The green core reaction chain indicates a common phase in the metabolic reactions leading to butyrate production. Here, H signifies redox reactions, CoA refers to coenzyme A, P denotes bound phosphate, Pi represents inorganic phosphate, and (B12) indicates enzymes dependent on vitamin B12.

## 2. Butyrate and Gut Health

### 2.1. Gut Health

The intestinal environment is regularly maintained by the actions of gut microbiota, which play some of the most vital roles such as energy balancing, prevention of mucosal infections, and the modulation of immune responses [28]. High-throughput DNA sequencing technologies have significantly advanced our understanding of the gut microbiota by providing detailed insights into the diverse community of microorganisms, particularly within the colon of the human gastrointestinal tract, without the need for traditional microbial culturing techniques [29]. Most of these species are grouped into two main phyla: Firmicutes and Bacteroidetes [30,31]. A diverse and well-balanced gut microbiota is needed for maintaining overall health [32]. In contrast, dysbiosis (highlighted by reduced diversity and changes in composition) is associated with conditions such as obesity, diabetes, and inflammatory bowel disease (IBD) [32,33,34]. Diet also influences the gut microbiota significantly, as different types of fibers produce varying effects on gut microbiota and, consequently, on gut well-being [35,36]. The gut microbiota is rich in synthesizing a variety of metabolites, containing SCFAs, polyamines, vitamins, tryptophan-derived compounds, and secondary bile acids, utilizing both undigested dietary substrates and endogenous materials [37]. These metabolites can serve as nutrients or substrates for other bacterial species in the colon, promoting additional metabolite production through interspecies cross-feeding [38]. It is believed that these metabolites can have both beneficial and detrimental impacts.

The mucosal protection and restoration processes of both the small and large intestines are highly effective and robust. These systems include a rapid turnover rate of epithelial cells, an efficient and well-functioning mucosal blood supply, a consistently maintained mucus layer that clings to the mucosal layer, and the presence of signaling peptides capable of initiating and enhancing the restoration processes [39]. With its single-layer structure, the intestinal epithelium serves as the body’s primary barrier and first line of defense against pathogens within the intestine, a defense mechanism primarily supported by its protective mucous layer [40]. The multipotent stem cells located within the crypts of the intestinal mucosa serve as the source for the specialized cell layer known as the epithelial monolayer, from which this layer is oriented and derived [41]. These multipotent stem cells give rise to four primary types of intestinal epithelial cells, each with specific functions vital for maintaining the integrity and functionality of the intestinal tract. The first type, absorptive enterocytes, is crucial for digesting and absorbing nutrients from the intestinal lumen into the bloodstream [42]. Secondly, goblet cells are responsible for producing and secreting mucins and trefoil peptides. These substances are essential for forming the mucus layer that protects and helps repair the epithelial surface [43]. Thirdly, enteroendocrine cells are specialized to produce and release peptide hormones into the bloodstream, which help regulate various aspects of digestive physiology [44]. Finally, Paneth cells secrete various antimicrobial peptides, such as cryptdins and defensins, along with digestive enzymes and growth factors. These secretions contribute to defending against pathogenic microorganisms and supporting the maintenance and renewal of epithelial cells [45]. The epithelial cells are connected by intercellular structures such as desmosomes, tight junctions, and adherens junctions. These structures create a physical barrier against the contents of the gut lumen and help maintain epithelial permeability [39]. Tight junctions are made up of a complex system that includes transmembrane proteins such as claudins, occludin, tricellulin, and junctional adhesion molecules [46,47]. Additionally, cytosolic scaffold proteins like zonula occludens and cingulin are integrated into this complex system [46]. Several antimicrobial peptides, including defensins and cathelicidins, as well as bacteriolytic enzymes like lysozymes, can be generated by both mucosal epithelial cells and Paneth cells [48,49]. This inherent ability of these cells to produce various antimicrobial substances is essential for protecting the mucosal tissue and the underlying gut stem cells [49]. By actively producing these defensive agents, these cells help maintain the integrity of the mucosal barrier and prevent harmful pathogens from invading the intestinal crypts, thus, playing a significant role in protecting and preserving the health of the intestinal environment [48,49,50,51]. The intestinal epithelium acts as a barrier between the gut lumen’s commensal organisms and the immune cells in the lamina propria [51]. Under normal conditions, homeostasis is maintained through complex immune interactions among the commensal microflora, the epithelial layer, and the subepithelial immune cells [51]. The lamina propria houses the gut-associated lymphoid tissue, which includes Peyer’s patches—a collection of lymphoid follicles containing various immune cells such as microfold cells, dendritic cells, T cells, B cells, intraepithelial lymphocytes, and macrophages [52]. 

### 2.2. Butyrate as a Postbiotic and Butyrate–Gut Health Relationship

It is evident that butyrate is generated in the gut environment by gut microbiota from molecules such as glutamate and more. Significantly, this magnificent metabolite has a prominent effect on gut health with various findings and reasons. Therefore, the gut microbiota species typically responsible for producing butyrate in humans are those belonging to the Firmicutes phylum, which includes species such as *Clostridium butyricum*, *Clostridium kluyveri*, *Faecalibacterium prausnitzii*, *Butyrivibrio fibrisolvens*, and *Eubacterium limosum* [27]. The primary butyrate producers are predominantly Gram-positive bacteria found within Clostridium clusters IV and XIVa of the Firmicutes phylum [53]. Among them, *Faecalibacterium prausnitzii* is the most prevalent in fecal samples, accounting for approximately 5% [54]. While most butyrate-synthesizing microorganisms belong to Firmicutes, studies have indicated that certain members of Actinobacteria, Bacteroidetes, Fusobacteria, and Proteobacteria can also synthesize butyrate [55]. In the human intestine, most of the microbial butyrate originates from carbohydrate metabolism, mainly facilitated by the butyryl-CoA: acetate CoA-transferase pathway (but) and butyrate kinase (buk) pathway. The but-pathway is particularly dominant in this process [56,57]. Thus, the majority of butyrate in the gut lumen is derived from non-digestible carbohydrates, mainly through the acetyl-CoA pathway. Furthermore, it has been suggested that the presence of butyrogenic bacteria in the colon, along with cross-feeding interactions between *Bifidobacterial strains* and *Faecalibacterium prausnitzii*, could potentially augment butyrate production [58].

Butyrate produced by the gut microbiome is quickly absorbed by the epithelial cells in the colon via passive nonionic diffusion or active carrier-mediated transport [59]. Butyrate, in its ionized state, is transported along the apical surface of colonocytes through active transport mechanisms involving H+-monocarboxylate transporter-1 (MCT1) and Na+-coupled monocarboxylate transporter-1 (SMCT1) [60]. Serving as a key SMCT1 transporter for butyrate uptake in gut epithelial cells, solute carrier family 5 member 8 (SLC5A8) is expressed predominantly at high levels in the apical membranes of the colon and ileum [60]. For butyrate’s mobilization, its action is facilitated by a carrier-mediated bicarbonate exchange framework, localized on the basolateral side [61]. Recognized as essential for upholding the epithelial barrier role and immunological homeostasis, intestinal microflora and their metabolites are significant factors. In an early study, it was observed that butyrate has properties that impact epithelial barrier function by acting as a key signaling substance for G-protein-coupled receptors (GPCRs) and nuclear factors [62]. Meanwhile, butyrate promotes T-cell-independent Immunglobulin A (IgA) excretion specifically in the large intestine by activating G-protein-coupled receptor 41 (GPR41) and G-protein-coupled receptors 109A (GPR109A), and even by inhibiting HDAC, thereby aiding in the restoration of epithelial integrity under inflammatory influences [63]. Butyrate also influences the gut microbial community by exerting control over IgA excretion and restricting the hyperresponsiveness of macrophages to large intestinal commensal microorganisms, thereby maintaining their profusion [63,64]. Acknowledging the significant immunomodulatory properties of butyrate, it physically enhances intestinal barrier function and acts as an anti-inflammatory agent by binding to SCFA-sensing GPCRs (GPR43, GPR41, GPR109A) [65]. Additionally, butyrate stimulates the immune system by modulating interactions between innate and adaptive immune cells within the gut mucosa. Moreover, in a study conducted on peripheral blood and colon tissues of 2,4,6-Trinitrobenzenesulfonic acid (TNBS)-induced colitis rats, alongside a control group, it was observed that butyrate administration enhanced Treg cell functionality and decreased interleukin-17 (IL-17) levels, along with Th17 cells, in the TNBS-induced colitis rats, while the control group exhibited minimal changes in these parameters [66]. In addition to the previously mentioned points, butyrate, through HDAC inhibition, can confine the production of pro-inflammatory cytokines such as IL-12 and IL-6 in colonic macrophages located in the lamina propria, while also limiting the antimicrobial activity of lipopolysaccharide (LPS)-stimulated macrophages by constraining nitric oxide production [64,67]. It is also important to highlight that the antimicrobial-inducing property of butyrate, specifically in macrophages, occurs independently of the GPCR pathway metabolites [68]. In this case, a performed study revealed that the expression of the antimicrobial protein calprotectin was highly increased, while the expression of the anti-inflammatory cytokine IL-10 decreased when the model macrophages were exposed to microbial butyrate [69].

Several studies have demonstrated the ability of butyrate to reduce nuclear factor kappa-light-chain-enhancer of activated B cells (NF-κB) activity in human colon-cell lines and in lamina propria mononuclear cells isolated from Crohn’s disease patients [70,71,72]. In addition, another study indicated that when the butyrate precursor, resistant starch, was administered orally to patients diagnosed with cholera, their health impairments improved significantly [73]. Moreover, the integrity and regulation of hypoxia-inducible factor are influenced by butyrate-producing bacteria in colonocytes, which upregulate mitochondria-dependent oxygen utilization due to butyrate production and its effects. When this factor is stabilized, it can regulate the tight junction protein claudin-1, MUC2 expression, and even the generation of the antimicrobial peptide beta-defensin-1 [74,75]. It is a notable aspect that this hypoxia-inducible factor could be deteriorated by the effects of butyrate [75]. Even a recent investigation, focused on these tight junctions specifically located in the gut epithelial tissue, found that butyrate maintains and controls the actin-binding protein synaptopodin. This protein is expressed at high levels in these tight junctions of the gut and is crucial for the integrity of the gut-barrier function [76].

General knowledge on gut health and dysbiosis is that their relationship significantly affects systemic and pancreatic diseases, including cancer, pancreatitis, and type 1 diabetes, by disrupting the intestinal barrier, promoting bacterial translocation, and aggravating inflammation and fibrosis [77,78]. In pancreatic and colorectal cancers (CRC), gut microbiota imbalances drive tumorigenesis through microbial diversity changes, elevated pro-inflammatory cytokines like IL-6, IL-10, IL-12, and IL-22, and mechanisms such as DNA alkylation by Escherichia coli and Fusobacterium nucleatum [79,80,81]. Beneficial microbes like Lactobacillus and Bifidobacterium counteract these effects by reducing inflammation and promoting antitumor activity [82,83]. Additionally, short-chain fatty acids (e.g., butyrate) regulate apoptosis and energy metabolism but are disrupted in CRC via the “Warburg effect” [84,85]. Dysbiosis also contributes to cardiovascular diseases, obesity, and inflammatory bowel conditions, while fecal microbiota transplantation shows promise in restoring balance and reducing inflammation, warranting further research [86,87,88].

Butyrate exhibits potent antitumor properties by modulating immune responses and the tumor microenvironment. It enhances innate immunity by promoting macrophage differentiation and reducing M2 macrophage polarization, which is associated with tissue remodeling and tumor progression [89,90]. Butyrate also supports adaptive immunity by stimulating Treg differentiation and antitumor CD8+ T cell responses, while increasing IgA-producing plasma cells in the gut assisting in cancer prevention [85,90,91]. Additionally, it mitigates tumor-associated inflammation by inhibiting pro-inflammatory cytokines like IL-6 and TNF-α, reducing the risk of tumorigenesis [92,93]. Butyrate induces tumor cell apoptosis via mechanisms such as the inhibition of the Wnt/β-catenin pathway, highlighting its role as a selective anticancer agent [94,95]. Expectedly, butyrate has significant cardioprotective effects through various mechanisms. It reduces pro-inflammatory cytokines such as TNF-α and IL-6 as mentioned before and enhances the protective Kruppel-like factor 2 (KLF2) pathway [96], thereby mitigating inflammation and oxidative stress. In atherosclerosis models, butyrate decreased lipid deposition and macrophage accumulation in plaques, improved gut permeability, and reduced aortic lesions [97,98].

The addition of butyrate to treatments in individuals with active ulcerative colitis (UC) has been associated with significant improvements in inflammatory markers [99,100,101,102,103]. In one study performed by Vernero et al., 12 months of butyrate-loaded microcapsule (BLM) administration resulted in sustained remission in most patients, as evidenced by low disease activity (MPS ≤ 2) and negative fecal calprotectin levels (<250 µg/g) [104,105]. In contrast, less than half of the control group maintained remission. While international guidelines suggest Escherichia coli Nissle 1917 (EcN) as the only alternative to mesalamine for maintaining UC remission [106], their findings support the potential of BLM as an effective therapy for these patients. Moreover, one of the recent studies conducted by Ozturk et al. [107] demonstrated that fecal SCFA levels, including acetic, propionic, and butyric acids, were significantly lower in patients with UC and Crohn’s disease compared to healthy controls, highlighting a potential association between reduced SCFAs and intestinal inflammation. In addition, a novel mode of action of butyrate in UC was developed and indicated its ability to alleviate neuronal loss in the enteric nervous system by reducing inflammation and oxidative stress, modulating immune responses, and enhancing gut barrier integrity [108]. These effects collectively assist in preserving neuronal function, contributing to improved gut motility and overall homeostasis in the context of chronic intestinal inflammation.

One study regarding the relationship between chronic kidney disease and butyrate interestingly displayed that *F. prausnitzii*-derived butyrate interacted with the GPR43 receptor to reduce renal inflammation, uremic toxin levels, and kidney pathology, as evidenced by decreased BUN and Scr levels, improved gut homeostasis, and suppressed macrophage infiltration in a CKD mouse model [109]. Therefore, it could be hypothesized that the butyrate–GPR43 axis represents a therapeutic pathway for this chronic kidney disease and highlights the importance of *F. prausnitzii* as a potentially potent probiotic.

The source of butyrate does not matter; it is absorbed by gut epithelial cells through diffusion. It then travels via the portal vein to the liver and enters systemic circulation [110,111]. Compared to the large intestine, the concentration of butyrate in the plasma is significantly lower and fluctuates as it enters systemic circulation, with only 2% making it into the bloodstream [112]. The remainder is distributed throughout other body parts. Other findings show that the presence of these butyrate-producing bacteria stabilizes intestinal homeostasis by creating an anaerobic milieu, which helps eliminate other opportunistic aerobic pathogen colonizations, such as Salmonella and *E. coli* [53,113]. Table 1 shows several works that have investigated the relationship between SCFAs (mainly butyrate) and the gut.

## 3. Butyrate’s Relation with Obesity

Obesity is a rapidly increasing condition seen in high numbers within various countries, posing a global risk to human life quality and lifespan. It is constantly being tracked by the World Health Organization (WHO) [114]. Day by day, the percentage of obesity is getting higher and becoming a more serious topic on health status of people, especially in countries like the USA, Turkey, and Saudi Arabia [115]. This increase raises concerns that the obese population may soon surpass the non-obese population. Diet, lifestyle, and lack of daily exercise are the main causes accelerating the onset of obesity. There are ongoing studies on this issue, with notable results seen in the utilization of butyrate for obese individuals.

Obesity is described as the extreme accumulation of fat, leading to severe levels of body mass with significant negative effects on health [116]. Body weight could potentially be maintained by butyrate, with reasonable speculation that it may influence energy utilization and/or general reduction in consumption, thus positioning butyrate at the core of energy homeostasis [117]. To understand how butyrate could influence this high accumulation of adipose tissue in the body, it is essential to examine the gut microbiome and its possible link to obesity. One remarkable thought that could serve as the causal bond between the obesity development and the environmental influences is actually the gut microbe resulting in the gut, suggesting that increasing evidence of the microbiota are involved in the energy controlling and substrate consumption and production [18]. Furthermore, it was previously discussed, dysbiosis—an abnormal status of the gut microbiome—results in disturbances in metabolism, inflammation, and immune function both locally and systemically, due to compromised gastrointestinal barrier integrity [118]. As a result, the intestinal microenvironment might close the space between caloric consumption and expenditure by metabolizing nutrients and controlling their entry into and retention within the body [118]. Among SCFAs, butyrate is especially recognized for its ability to mitigate obesity and metabolic disorders prevalent in many societies and this effect is achieved through the precise regulation of hormones and mediators that maintain energy balance [7]. Findings suggest that reduced levels of microbes responsible for butyrate production in humans are linked to a heightened risk of metabolic disorders, highlighting butyrate’s significant role in easing obesity-related metabolic imbalances [118]. Thus, investigating the microbial origins of this fermentation byproduct and assessing how dietary changes and gut function might impact its levels and rates of synthesis is essential.

Several new efforts have recently been technically explored to improve the taste and delivery of butyrate in the digestive tract, while the inclusion of sodium butyrate supplements in the diet is one of the most prominent clinical and animal studies primarily targeting obesity and diabetes [119]. This technique popularly used to delay the release of sodium butyrate in the gut tract involves encasing it in cellulose-based capsules [120]. Butyric acid is recognized as a safe compound, supported by pharmacological and clinical research, as therapeutic doses ranging from 150 to 300 mg have shown no abnormal side effects or clinical abnormalities [121]. Additionally, no traces of abnormal effects were observed even at high doses of up to 2000 mg per day [12,122]. Additionally, recent studies suggest that butyrate may have a significant role in promoting fatty acid oxidation, as indicated by critical changes in serum triglyceride levels and the respiratory exchange ratio in animals on a high-fat diet (HFD) compared to control animals receiving only a HFD [123,124,125]. Moreover, another study found that butyrate significantly lowered lipid levels in brown adipose tissue (BAT), with a somewhat lesser effect observed in the both liver and muscle tissues [126].

In several studies, two different methods demonstrated a common reduction in body weight changes: either as an additional supplement in food [123,127,128] or administered directly via gavage [124,129,130], including mass loss and gain, and fluctuations in fat ratio, observed in rodents on a HFD compared to controls that only received HFD feeding. The results suggest that butyrate could potentially play a significant role in the treatment or even prevention of diet-induced obesity (DIO). The administration of sodium butyrate via intraperitoneal injection has shown positive and consistent results in this regulatory function, leading to a significant decrease in body weight gain in rodents over ten consecutive weeks when they received a butyrate-supplemented diet [131]. The addition of butyrate to daily diets reveals numerous metabolic features and regulatory effects, including crucial prevention of HFD-induced obesity and related disorders. This has primarily been observed in animal models [132,133,134].

In a study conducted by Pelgrim et al. [133], the effects of a HFD on low-density-lipoprotein receptor knockout Leiden mice of different age ranges were examined, focusing on metabolism and the role of adipose tissue in the regulation and outcomes of butyrate. The results indicated adipocyte hypertrophy and an increased number and activity of proinflammatory adipokines, which therefore identified the HFD as a source of enhanced inflammation. The addition of butyrate to the diet resulted in a significant overall reduction in body weight and fat, independent of daily calorie consumption, as evidenced by the adipocytes becoming firmer and smaller. However, this effect was primarily observed in older adults as the sample type. Among these older adults, particularly those around 65 years old, the reduction in adipocyte size was harder to observe. Interestingly, despite the lack of significant reduction in adipocyte size, insulin levels decreased, and inflammatory activity also declined. Therefore, the effects of butyrate observed in this study highlight its potential to refine obesity-related metabolic disorder and inflammation, particularly in older adults with varying levels of activity.

Additionally, supplementing mice with sodium butyrate revealed its protective role against liver and pancreatic abnormalities. This was shown by a significant reduction in liver steatosis and a marked decrease in fat accumulation in the pancreas in another study examining sodium butyrate’s effects on animal models [134]. The efforts in this study regarding the treatment resulted in fluctuations in insulin resistance, as well as stabilization of insulin release. Consequently, β cells visibly expanded in size and corresponded with more stable blood sugar level regulation. Butyrate also improved gut health in mice, particularly by enhancing gut integrity through elevated claudin-1 levels and reduced gut permeability. In summary, butyrate has the potential to enhance gut health and could, therefore, help address metabolic issues

In one of the further human studies, the administration of 4 g of sodium butyrate daily to obese individuals diagnosed with various metabolic disorders caused enhanced innate immune memory for LPS and increased IL-6 and Pam3CSK4-induced TNF-α responses, while also reducing oxidized low-density lipoprotein (oxLDL) levels. Considering the significance of these anti-inflammatory and immunomodulatory effects, butyrate supplementation could be a novel therapy to slow the progression of vascular wall inflammation and prevent the development of atherosclerosis, which is closely related to obesity [135]. This anti-inflammatory effects of butyrate have been recognized, particularly due to its role as an epigenetic modulator through the inhibition of HDAC [136]. According to the available data, butyrate may be considered a promising strategy for enhancing sustained energy balance.

The obesity is thought to be a primary risk factor for causing the development of type 2 diabetes [117]. Although the supplementation of butyrate can not only aid in avoiding the onset of diabetes by mitigating obesity, butyrate administration as a supplement can also act as an HDAC inhibitor, thereby showing antidiabetogenic features [16]. As thoroughly discussed in previous sections, the role of butyrate in maintaining metabolic homeostasis and facilitating communication between the gut microbiota and metabolism is immensely important. Due to the link between obesity and consequently more advanced conditions, such as type 2 diabetes, it is now recognized as the most prevalent metabolic disorder in the human population [137].

Type 2 diabetes is an immensely common disorder in the human population, characterized by the pancreatic β-cells losing their functionality and the development of resistance to insulin in specific target tissues [138]. Consequently, it is defined as comparative insulin insufficiency [138]. This immensely common disorder in the human population, type 2 diabetes, is characterized by the pancreatic β-cells losing their functionality and the development of resistance to insulin in specific target tissues, thus, being defined as comparative insulin insufficiency [138]. On top of that, several studies have underscored the strong connection between the gut microbiota and type 2 diabetes. Within these studies, the significant impact of butyrate on this metabolic disorder has been accentuated. On top of that, several studies have underscored the strong connection between the gut microbiota and type 2 diabetes. Within these studies, the significant impact of butyrate on this metabolic disorder has been accentuated [139,140]. One of the fundamental studies showed that the complaint rate in newly diagnosed patients with type 2 diabetes was positively associated with body mass index, fasting plasma glucose, and being female, with specific symptoms like dry mouth, thirst, and stomach pain linked to fasting plasma glucose [141]. Additionally, symptoms such as shortness of breath, swollen ankles, and heartburn increased with body mass index, while obesity-related symptoms have often been underestimated [141]. Insulin resistance, one of the hallmarks of type 2 diabetes, has been linked to metabolic dysfunctions that contribute to obesity symptoms, such as increased adiposity and chronic low-grade inflammation.

A study conducted by Sanna and her colleagues [142], integrated fecal microbiota metagenomic data with human genome sequencing data. The results indicated that high levels of butyrate production, influenced by the host’s genetic makeup, were elucidated through this integrated approach. This increased butyrate production could be linked to improved insulin feedback to an oral glucose challenge in normoglycemic patients. In addition, another study explored the variation in the gut microbiota during non-nocturnal states [143]. It was revealed that this variation was disrupted in patients diagnosed with type 2 diabetes. Specifically, the rhythmicity of various bacterial species, including Roseburia and F. prausnitzii, was found to be irregular. This lack of rhythmicity is believed to be significant for risk assessment and forecasting of type 2 diabetes.

Many epigenetic modulations in various organs have been identified in connection with type 2 diabetes, with a deeper hypothesis suggesting that butyrate, produced by butyrate-producing bacteria, is a key determining factor [144]. From the gathered information, it is possible to explore type 2 diabetes-related impairments influenced by butyrate levels. HDAC activity was found to be increased, leading to elevated ROS expression, especially when butyrate levels in the gut were altered, along with changes in colonic permeability in a non-obese animal model [145].

The previously mentioned HDACs and histone acetyltransferases (HATs) regulate cellular acetylation and secure chromatin [146]. This function of these enzymes explains why they are associated with gene expression and transcriptional changes [146,147]. Therefore, HDACs are key elements in the development of type 2 diabetes, as they significantly influence both lipid and glucose metabolism. The connection between butyrate, HDACs, and type 2 diabetes suggests a potential novel clinical strategy in this context [148,149]. Some other studies also investigated the association between HDAC inhibition and butyrate supplementation. In one study [131], sodium butyrate was administered alongside metformin to explore its effects on diabetes-related disorders in rats. The findings showed that both sodium butyrate and metformin significantly reduced fat accumulation, dyslipidemia, and insulin resistance. Additionally, glucose management improved, and histological damage in specific liver and pancreatic tissues was mitigated. When sodium butyrate and metformin were used together, HDAC activity was suppressed, and gluconeogenesis levels were specifically reduced due to changes in forkhead box protein O1 and glucagon expression. Future research should carefully investigate the mechanisms behind the beneficial effects of sodium butyrate and metformin, particularly focusing on how HDAC inhibition contributes to diabetes management. Moreover, combining sodium butyrate with other therapeutic agents might enhance treatment effectiveness and offer new strategies for managing type 2 diabetes, as highlighted by this study [131].

Meanwhile, Gao et al. studied mouse models subjected to a high-fat diet (HFD) [150]. The mice that received 400 mg/kg of butyrate orally exhibited improved glucose tolerance. Specifically, there was a relative increase in the expression levels of phosphorylated adenosine monophosphate kinase (AMPK) and glucose transporter-4 in adipose tissue. Additionally, some of the shifts in the gut microbiota induced by the HFD were reversed [150]. In another study, mouse models were used to investigate the effects of an HFD, which contains 5% butyrate in their HFD as a key regimen [123]. The results of this treatment indicated increased AMPK activity along with improved insulin sensitivity. Additionally, the intervention caused significantly higher energy expenditure, enhanced mitochondrial biogenesis, and stimulated adaptive thermogenesis in BAT [123]. Ultimately, one of the most recent studies explored the relationship between SCFAs, specifically butyrate, and their impact on obesity, glucose regulation, and insulin secretion in individuals with metabolic disorders, especially type 2 diabetes and obesity [151]. In the study, 12 individuals with obesity and type 2 diabetes were selected, along with eight healthy participants. The researchers assessed butyrate concentrations, along with other SCFAs, metabolic indicators, and body composition. These evaluations took place before and after a 12-month regimen that included a protein-rich diet, moderate exercise, and standard medication. The results showed significant reductions in body mass index (BMI), visceral fat area, fasting plasma glucose, insulin resistance, and other metabolic markers in the type 2 diabetes group following the intervention. Additionally, levels of butyrate and isobutyrate increased after substantial weight loss. The observed correlation between butyrate and indicators of obesity, such as BMI, visceral fat area, and glucose metabolism, suggests that butyrate, along with other SCFAs, could play a beneficial role in managing metabolic health and obesity. These findings highlight the potential for butyrate to be used as a therapeutic agent for obesity and type 2 diabetes in the near future [151]. Nevertheless, further research is needed to comprehensively understand the actual mechanisms behind these effects and their potential applications for improving human metabolic health. If additional studies are conducted to fully understand the side effects and relevance of butyrate to human physiology, it could pave the way for butyrate and other SCFAs to be utilized in the pharmaceutical and supplement industries. Last but not least, it is evident that dietary resolution is a health concern. Type 2 diabetes has even more catastrophic effects in patients with hypertension, and in this case, butyrate also has an effect against hypertension [152,153], which is often seen in older individuals. One study found that higher fecal butyrate levels were inversely associated with hypertension in overweight and obese cancer survivors, with increases in butyrate over a year linked to lower blood pressure [152]. The findings suggest that butyrate may be a potential target for blood pressure-lowering interventions, though further research is needed to determine its clinical significance.

The observed link between type 2 diabetes and a major decline in butyrate-producing bacteria has prompted growing interest in investigating the therapeutic possibilities of butyrate as a current and future treatment option for type 2 diabetes. Although preclinical research has yielded generally encouraging findings, clinical trials in humans have demonstrated beneficial effects predominantly over the short duration, with significant constraints emerging upon longer evaluation. To advance knowledge in this specific field, further research is absolutely essential to identify the gut microbiota factors that could impact butyrate production in type 2 diabetes patients. Such investigations should consider both dietary influences and the unique composition of an individual’s intestinal microbiota. Addressing these elements will provide critical insights into how butyrate therapy could be optimized for managing type 2 diabetes in a personalized and more sustained manner.

Table 2 shows several studies on butyrate-induced models of common metabolic disorders.

## 4. Butyrate as a Neurological Health Enhancer

There is ongoing global interest in the gut microbiota and its relevance to the nervous system. Therefore, the physiological role of the central nervous system, and consequently both human and animal behavior, is influenced by the existence, metabolism, and activity of microorganisms [154]. The relationship between our gut microbiota and nervous system is a large part of the gut–brain axis that has attracted increasing interest in recent years [155]. This network includes the central nervous system, incorporating the enteric nervous system, the autonomic nervous system, the neuroendocrine system, the neuroimmune system, and the gut microbiota. These components collectively interact to form a comprehensive communication system that links the gastrointestinal tract with the brain, facilitating complex regulatory and feedback mechanisms [156]. The occurrence of balance disruption in gut microbiota may cause changes in the brain, specifically in how it functions. These changes could be associated with various neurological disorders, such as Autism Spectrum Disorder, Parkinson’s disease, and Alzheimer’s disease [157,158,159]. Even though the relationship between the gut microbiota and alterations in the brain is not yet fully understood, there are various pathways that are thought to be involved in this complex and multifaceted connection [155]. It is well established that gut bacteria possess significant metabolic capability, and several microbe-related metabolites are able to enter the bloodstream and potentially penetrate the blood–brain barrier, allowing them to influence brain function and possibly contribute to neurological health or disease [155]. These so-called microbe-oriented neurotransmitters, primarily gamma-aminobutyric acid (GABA) and serotonin, are believed to have the ability to modulate the immune response, alter epigenetic markers, and generate bioactive compounds [160,161,162].

Butyrate, in relation to the brain, exerts beneficial effects indirectly by enhancing the overall cohesion and stability of the intestinal barrier, which plays a critical role in maintaining gut health. It is widely recognized that the intestines are often referred to as the “second brain” of the human body, highlighting their significant influence on overall health and neurological function. In addition to these benefits, butyrate modulates the immune system and stimulates the release of glucagon-like peptide-1 and peptide YY in the periphery by activating GPCRs in enteroendocrine cells, contributing to improved metabolic and gut–brain signaling processes [163,164]. To date, butyrate has been identified as an activator of GPCRs. These previously mentioned receptors include GPR43, which has been renamed free fatty-acid receptor 2 (FFAR2), GPR41, now referred to as free fatty-acid receptor 3 (FFAR3), as well as GPR109a and GPR164, which has been renamed OR51E1 and is known as Olfr558 in mice. [165]. This topic emphasizes the intricate interactions between butyrate and these specific GPCRs, further emphasizing its role in various physiological processes mediated through receptor signaling pathways.

Butyrate plays a significant role in interacting with these GPCRs, particularly FFAR3 and GPR109a, thereby influencing both energy metabolism and inflammatory processes [166]. FFAR3, which is activated by butyrate, has been found to regulate the sympathetic nervous system and intestinal gluconeogenesis, suggesting its importance in both neural and metabolic functions [167]. Meanwhile, GPR109a is another key receptor activated by butyrate and expressed in colon cells and microglia, where it mediates strong anti-inflammatory effects [155]. The activation of GPR109a in colon cells has been linked to the induction of apoptosis, particularly in colon cancer cells, highlighting its potential role in cancer therapy [168]. Additionally, in models of Parkinson’s disease, GPR109a activation has been shown to reduce neuroinflammation, further illustrating its neuroprotective properties [169,170]. Interestingly, although β-hydroxybutyrate, another metabolite, acts as an antagonist of FFAR3, it functions as an agonist for GPR109a, which points to its potential utility in anti-inflammatory therapies [171]. This dual action underscores the therapeutic value of targeting these receptors for various conditions, including inflammation and neurodegenerative diseases.

On the other hand, the relationship between SCFAs and butyrate in SCFA receptors is quite significant due to their roles in immunity, including the management of neuroinflammation, the host energy-metabolic processes, and the neuroendocrine control of bodily actions and behavior. Major psychiatric disorders, such as depression, have demonstrated a remarkable reduction in mental illness-like behavior in animal models when histone hyperacetylation is stimulated by butyrate, underscoring its considerable impacts [172]. Additionally, the suppression of HDAC is strongly associated with autism spectrum disorders at the cellular basis, which are marked by reduced neural inhibitory signaling, particularly GABAergic signaling [173]. Since it is well established that butyrate possesses the important ability to cross the blood–brain barrier and acts as an inhibitor of HDACs, it can also alter the gut activity by influencing macrophages and lowering the synthesis of pro-inflammatory cytokines in response to LPS through its HDAC inhibitory activity [174]. Consequently, this modulation enhances systemic defense, with a particular benefit to brain defense mechanisms, underscoring the broader implications of butyrate’s effects on both gut and brain health [174,175].

A study primarily highlighted the use of butyrate in Black and Tan BRachyury (BTBR) mice as a model to investigate autism-related features [176]. It was found that the models, when administered a low dose of 100 mg/kg, were insufficient to produce measurable changes in histone acetylation, particularly in the prefrontal cortex. However, although the changes in histone acetylation were not significant, other social challenges within the model animals were alleviated. In another study, Gagliano and colleagues aimed to investigate the stress-like effects of sodium butyrate specifically targeting the hypothalamic–pituitary–adrenal (HPA) axis. Their findings revealed that administering a higher dosage of sodium butyrate, at 1200 mg/kg, was effective in inducing a stress-like response within the HPA axis. In contrast, a significantly lower dosage of sodium butyrate, at 200 mg/kg, was found to be insufficient to trigger the same stress response, demonstrating a clear dose-dependent effect [177]. An alternative study emphasized sodium butyrate’s role as an HDAC inhibitor and its potential as a novel neuroprotective compound against stroke and neurodegenerative disorders [23]. The experiments demonstrated that brain damage in mouse models was effectively attenuated, thus, preserving brain integrity with sodium butyrate administration. However, the use of sodium butyrate was found to be quite challenging in laboratory conditions due to potential toxic effects. In this context, it is believed that further developments are needed to avoid toxicity while maintaining its neuroprotective properties, so that butyrate can present its full potential for therapeutic use in patients suffering from neurological disorders, ultimately aiding in neurological recovery. A different study was performed on mouse models carrying Alzheimer’s disease [178]. The models were subjected to a diet composed solely of a rich fiber source, fructans, to reestablish gut modifications. After the specific diet, the levels of butyrate-producing bacteria in the microbiota significantly increased, along with the amount of butyrate in the fecal composition. The mice exhibited improved cognitive and spatial memory, as well as reduced anxiety. These changes were significant compared to the control groups, which were given either antibiotics or a regular daily diet [178]. A study conducted by Matt and his teammates found a significant correlation between the administration of sodium butyrate and its effects observed in both young and old mice groups [19]. The research demonstrated that when sodium butyrate was introduced to these mouse models, there was a notable and drastic reduction in the expression levels of IL-1β genes within both the microglia and the hippocampus. Additional studies on Alzheimer’s disease have shown that the introduction of sodium butyrate supplementation resulted in the induction of expression levels in learning-related genes and the reestablishment of histone acetylation. [179,180]. The details of these studies reveal that sodium butyrate administration to the models had significantly positive influences on contextual memory in transgenic models, even at the terminal phases of Alzheimer’s disease. However, such significant outcomes were not observed in wild-type models. In addition to globally threatening neurological disorders, Parkinson’s disease was also subjected to sodium butyrate treatment in mouse models for a study [181]. The results indicated an alleviation of motor impairments, an increase in dopamine levels in the striatum, and a reduction in stress caused by reactive oxygen species (ROS) and neuroinflammation. Interestingly, global acetylation levels of histone H3 and brain-derived neurotrophic factor (BDNF) were increased [181]. Another study similarly found that sodium butyrate supplementation in dopaminergic cells helped hinder the onset of apoptosis and facilitated DNA damage repair in vitro [182]. Since sodium butyrate has demonstrated a variety of effects [23,155,169,183], including neuroprotection in Parkinson’s disease models as well as mitigation of cisplatin-induced hearing loss, its potential therapeutic benefits extend across different types of conditions. Meanwhile, Kratsman et al. investigated the effects of sodium butyrate administration in autistic mouse models [176]. The team found that sodium butyrate administration specifically increased the expression levels of inhibitory genes in the frontal cortex while suppressing excitatory genes, thereby inducing an overall increase in inhibitory gene expression. The experiments suggested that low doses of sodium butyrate (100 mg/kg) were not a significant parameter, as the differences in results between dosage treatments were not substantial. Despite this, social impairments were still interestingly mitigated [176].

Alongside this, Huntington’s disease is also demonstrating its significant influence as another highly risky neurological disorder that poses considerable challenges. Extensive studies oriented towards Huntington’s disease have been conducted across various animal models, aiming to understand its effects more comprehensively. The results from these studies indicated that the administration of supplements such as sodium butyrate and phenylbutyrate—phenylbutyrate being an analog of butyrate—led to the restoration of histone acetylation. As a consequence of these findings, the level of apoptosis in neuronal cells was notably reduced, and, as a result, the life expectancy of the mice used in the studies was observed to have increased [184,185]. More substantially, additional studies uncovered that sodium butyrate has the ability to replenish Complexes I, II, III, and IV, as well as counteract the inhibition of the Krebs cycle caused by amphetamines and ouabain in animal models exhibiting mania [186,187,188]. This feature may, therefore, singularly assist in fixing the disease-oriented mitochondrial abnormality in the brain.

Several butyrate-producing bacteria, including *Faecalibacterium prausnitzii* and *Roseburia intestinalis*, were found to be significantly elevated in models, such as pigs when fed a diet abundant in arabinoxylan. This diet was compared to one consisting of a standard and highly resistant starch percentage. The results indicated that the diet rich in arabinoxylan had a considerably greater impact on the levels of these beneficial bacteria compared to both the resistant starch diet and the standard diet [189,190].

In a key study, Amoldussen and their colleagues [132] attempted to observe whether an HFD influenced changes in mid- and late-adult low-density lipoprotein-receptor knockout mice while consistently supplementing them with butyrate. They found that an HFD in middle-aged mice resulted in elevated neuroinflammation, as evidenced by the increased activation of microglia, the brain’s immune cells. This rise in inflammation was related to a decrease in functional connectivity within the somatosensory cortex and hippocampus, regions known to be essential for sensory processing and memory [132]. These findings by Amoldussen and their team suggest a highly potential link between dietary habits, brain inflammation, and reduced brain connectivity. Late-adult mice, in comparison to middle-aged mice, did not show a significant impact from the additional butyrate procedure. This lack of effect is expected because the circulatory system in late-adult mice is undergoing permanent senescence. This study revealed that butyrate influences gut microbiota linked to liver fibrosis and neuroinflammation, demonstrating its potential in reducing cognitive impairment associated with obesity. However, its efficacy is more significant in middle age and less effective in late adulthood due to accelerated senescence.

**Figure 3:**
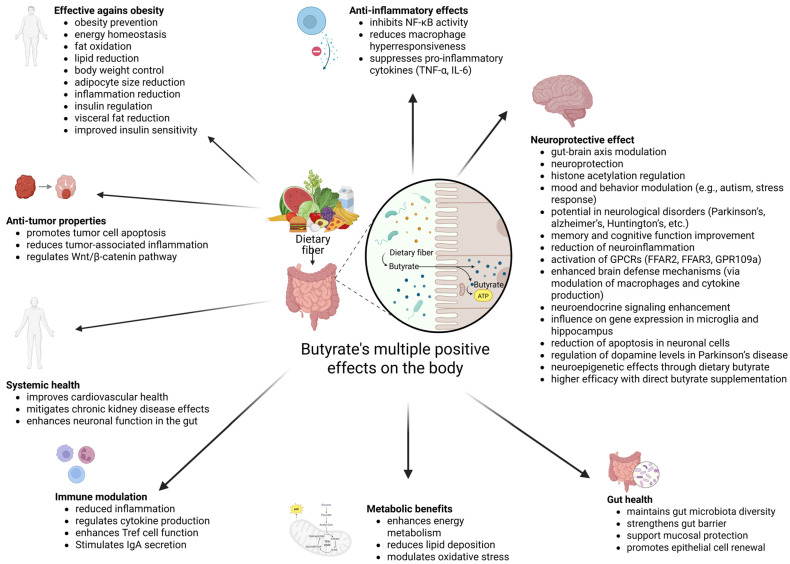
A diagram displaying the multiple positive effects of butyrate on body health [3,32,60,65,92,108,138,142,145,151,156,157,171,174].

Sodium butyrate demonstrates markedly superior immunological and neurological benefits when compared to butyrate derived from a high-fiber diet. This amplified effectiveness is likely due to the direct administration of sodium butyrate in higher doses, while dietary butyrate generally affects brain function through gut-related pathways. Current literature emphasizes the importance of the gut–brain axis and nutrition in both disease development and treatment. To better understand the potential benefits, future research should explore the neuroepigenetic effects of dietary butyrate more thoroughly. The dose–response relationship should be examined more precisely in future studies to determine how much specific butyrate actually reaches the brain. Such research could reveal the therapeutic potential of butyrate more clearly. Additionally, the synergistic effects of various types of dietary fiber and fermented foods should be considered, as their combination could significantly increase butyrate levels and optimize its benefits. In Table 3, we can see some investigations that indicate the relationship between butyrate and neurological effects in models. Figure 3 presents a diagram displaying the multiple positive effects of butyrate on body health.

## 5. Conclusions

Future research on butyrate should analyze its potential benefits beyond gut health. It is important to explore how butyrate might offer therapeutic advantages for chronic conditions such as cardiovascular diseases, diabetes, and neurodegenerative disorders. Investigating butyrate’s comprehensive systemic effects could reveal its role in regulating the immune system and inflammation, potentially improving the management of these diseases. A more detailed understanding of how butyrate interacts with our genetics and gut microbiota could contribute to more personalized health plans, including customized dietary recommendations and treatment guidelines designed to individual needs. Looking ahead, attention should also be directed toward developing therapies and functional foods based on butyrate. It is important to study how environmental and lifestyle factors influence butyrate production as well. Integrating this research with global health initiatives will help us gain a thorough understanding of butyrate’s role across various populations and develop effective dietary guidelines.

It is particularly significant to explore how butyrate affects obesity and type 2 diabetes. Research should focus on how butyrate impacts metabolic health, including its interactions with gut microbiota, dietary factors, and individual genetic differences. Performing clinical trials in a wider fashion could help evaluation of the long-term benefits and safety of butyrate supplementation. This possible approach could offer valuable perceptions and might eventually give rise to its use in clinical practice as a sustainable treatment option.

For neurological health, future research should focus on how butyrate interacts with the renowned gut–brain axis. It is crucial to understand how butyrate influences neurotransmitter levels, neuroinflammation, and histone acetylation. Investigating how different dosages and forms of butyrate affect conditions like Alzheimer’s and Parkinson’s diseases could help polish its use in therapy. Additionally, looking into how dietary fibers and fermented foods impact butyrate production and its delivery to the brain might disclose new strategies for improving cognitive function and neuroprotection, causing more effective treatments for neurological disorders.

Even recent studies have concluded that butyrate inclusion plays a key role, one example being that halloysite nanotubes (HNT) enhance the mechanical strength, crystallinity, and biocompatibility of polyhydroxybutyrate (PHB)-based scaffolds for cartilage regeneration. This highlights butyrate’s bioactive properties in a different field, where its integration into HNT-modified PHB scaffolds could further improve tissue regeneration and chondrocyte proliferation, having a significant impact on biomedical advancements [191]. Another area poised for improvement through butyrate studies is environmental rejuvenation efforts. Recent advancements have demonstrated that PHB-based composites reinforced with cellulosic fibers can enhance mechanical properties and structural integrity for agricultural applications. Future research could explore incorporating butyrate into these bio-based materials to further improve their biodegradability and functional benefits [192]. These new works have made butyrate’s multifaceted role even more prominent, and day by day, its applications are expanding, offering more potential in the future.

In conclusion, thoroughly exploring butyrate’s systemic effects, therapeutic potential, and interactions with various biological and environmental factors will be another major key to exploiting its benefits for many metabolic health conditions and improving overall public health.

## Figures and Tables

**Table 1:** Several studies that show relations between SCFAs (mainly butyrate) and the gut.

Supplement	Microorganism	Outcome(s)	Reference
Natural butyrate producers analyzed	*Firmicutes* (dominant), *Fusobacteria*, *Bacteroidetes*	Identification of four main butyrate production pathways (acetyl-CoA, glutarate, 4-aminobutyrate, lysine), conservation of specific genes, and functional predictions for microbial butyrate synthesis.	[55]
Butyrate	*Coprococcus*, *Roseburia*, *Lachnospira*, *Butyricimonas*	Reduced circulating butyrate levels and decreased butyrate-producing bacteria abundance in developed late-onset preeclampsia women.	[56]
SCFA	*Coprococcus* (correlated with SCFA levels in controls)	Lower acetate and propionate levels trend; significant decrease in butyrate.	
None	*Coprococcus*, *Bifidobacterium*	Significant negative correlation between butyrate abundance and fasting triglyceride levels.	
Butyrate-producing community analysis	*F*. *prausnitzii*, *Roseburia* sp., *E*. *rectale*, *Acidaminococcus* sp.	Demonstrated high abundance of butyrate-producing communities (5–26%) in patients after ileostomy takedown.	[57]
16S rRNA gene analysis	*E. hallii*, *E. rectale*, *Subdoligranulum* sp., *Anaerostipes* sp.	Supported functional gene data but showed discrepancies in detecting certain butyrate producers, like *Eubacterium* sp.	
*F. prausnitzii* alone or co-culture with *Bifidobacterium*	*F. prausnitzii*	Improved viability and gut colonization of *F. prausnitzii*, alleviated colitis symptoms in DSS (dextran sodium sulfate)-induced colitis model.	[58]
*Bifidobacterium* (*B. catenulatum*, *B. animalis* strains)	*Bifidobacterium* strains (*B. catenulatum*, *B. animalis*)	Enhanced growth and butyrate production of *F. prausnitzii*, improved intestinal delivery in co-culture.	
*F. prausnitzii* and *Bifidobacterium* co-culture	*F. prausnitzii*, *Bifidobacterium*, *A. muciniphila*	Increased gut colonization, particularly of *F. prausnitzii*, and altered microbiome composition (higher *A. muciniphila* abundance).	
Butyrate	-	Transported across equine colonic luminal membrane via MCT1 (Monocarboxylate transporter 1) by an electroneutral, H+-symport mechanism; upregulated by increased luminal butyrate concentrations.	[59]
Acetate, Propionate	-	Inhibited butyrate transport via competition or shared transport protein MCT1; may affect SCFA absorption under conditions of excessive lactic acid in the gut.	
SCFAs	-	Affect colonic cell proliferation and function as an energy source for intestinal epithelial cells.	[60]
Butyrate	*Ruminoclostridium*, *Roseburia*	Activation of the Aryl hydrocarbon receptor (AhR) pathway in intestinal epithelial cells.	[62]
Butyrylated high-amylose maize starch diet	Increased IgA production and enhanced mucosal barrier function in the colon during inflammation	Fecal microbiota from SPF (specific pathogen-free) mice (via transplantation).	[63]
n-Butyrate	-	Reduced secretion of IL-6, IL-12p40, and nitric oxide (NO) in bone marrow-derived macrophages (BMDM) and colonic lamina propria macrophages.	[64]
Tributyrin	*Citrobacter rodentium*	Mitigated acute antibiotic- and ethanol-induced gut microbial disturbances; reduced Gram-negative bacterial growth.	[65]
	*Roseburia hominis*	Increased abundance of *Roseburia hominis* in antibiotic and ethanol-exposed mice.	
Butyrate-treated rats	-	Ameliorated weight loss, increased colon inflammation, and lower Neurath scores.	[66]
Butyrate	-	Human UC patients: Significantly lower net concentration of butyric acid compared to healthy controls. TNBS-treated rats: Lower butyric acid and total SCFA concentrations compared to control rats. Butyrate administration in rats: Increased fecal concentration of butyric acid, total SCFA, and percentage of butyric acid.	[67]
Butyrate	*C. rodentium*, *Salmonella*	Enhanced bactericidal function of colonic macrophages and reduced systemic bacterial dissemination in *C. rodentium* and *Salmonella* infections.	[67]
Butyrate	-	Suppresses NF-κB activation in HT-29 cells by stabilizing IκB-α and increasing p100 levels, potentially through reduced proteasome activity.	[68]
Butyrate	-	Suppressed mucosal inflammation and constitutive NF-κB p50 dimer activity in HT-29 cells.	[71]
Butyrate	-	Enhances intestinal epithelial barrier formation (increased transepithelial electrical resistance over 72 h)	[73]
Butyrate	Microbiota-derived butyrate	Stabilizes hypoxia-inducible factor-1, regulates gut homeostasis genes, increases 2-OG (2-oxoglutarate) levels.	[75]
Butyrate	-	Significant promotion of intestinal barrier function, increased transepithelial electrical resistance, accelerated wound closure, and upregulation of synaptopodin expression (both mRNA and protein levels).	[76]

Abbreviations: 2-OG, 2-oxoglutarate; AhR, Aryl hydrocarbon receptor; BMDM, bone marrow-derived macrophages; DSS, dextran sodium sulfate; IgA, Immunoglobulin A; IκB-α, inhibitor of kappa B alpha; IL-6, Interleukin-6; mRNA, messenger RNA; MCT1, Monocarboxylate transporter 1; NF-κB, nuclear factor kappa-light-chain-enhancer of activated B cells; NO, nitric oxide; SCFA, short-chain fatty acid; SPF, specific pathogen-free; TNBS, 2,4,6-Trinitrobenzenesulfonic acid; UC, ulcerative colitis.

**Table 2:** Several studies on butyrate-induced models of common metabolic disorders.

Focus Area	Model	Findings	Reference
Energy Expenditure and Thermogenesis	HFD mice (5% butyrate diet)	Increased AMPK activity, improved insulin sensitivity, higher energy expenditure	[123]
Diabetes Management	Rat models	Reduced fat accumulation, improved glucose management, lowered HDAC activity	[131]
Obesity and Metabolic Disorders	HFD in LDLR-KO mice	Reduced body weight, smaller adipocytes, lower inflammation, insulin regulation	[133]
Liver and Pancreatic Health	HFD animal models	Reduced liver steatosis, less pancreatic fat, improved β-cell function and insulin stability	[134]
Inflammation and Atherosclerosis	Obese individuals	Enhanced immune memory, reduced oxidized LDL, potential in vascular inflammation treatment	[135]
Type 2 Diabetes and Genetics	Human metagenomic and genomic data	High butyrate levels linked to better insulin response	[141]
Type 2 Diabetes	Human microbiota study	Disrupted bacterial rhythms in diabetic patients; potential predictive marker	[142]
Type 2 Diabetes and Epigenetics	Various animal models	Butyrate altered HDAC activity, reduced oxidative stress and improved gut permeability	[143,144]
Glucose Metabolism and Obesity	HFD mice	Improved glucose tolerance, increased AMPK and GLUT-4 expression	[149]
Obesity and Type 2 Diabetes	Obese and diabetic individuals	Higher butyrate levels linked to lower BMI, visceral fat, and better glucose metabolism	[150]

Abbreviations: AMPK, adenosine monophosphate kinase; BMI, body mass index; GLUT-4, glucose transporter-4; HDAC, histone deacetylase; HFD, high fat diet; LDL, low-density lipoprotein; LDLR-KO, low-density lipoprotein receptor knockout.

**Table 3:** Some of the studies indicating the relationship between butyrate and neurological effects in models.

Focus Area	Model	Findings	Reference
Neuroprotection in Stroke	Mouse models	Sodium butyrate mitigated brain damage and preserved brain integrity in stroke models, although toxicity was a challenge.	[23]
Autism Spectrum Disorder	BTBR mice	Low dose of sodium butyrate (100 mg/kg) showed no significant histone acetylation changes but alleviated social impairments.	[173]
Autism Spectrum Disorder (Gene Expression)	Autistic mouse models	Sodium butyrate increased inhibitory gene expression in the frontal cortex, but low doses (100 mg/kg) had minimal effect on social impairments.	[173]
HPA Axis Stress Response	Mouse models	Higher dose of sodium butyrate (1200 mg/kg) induced a stress-like response in the HPA axis; lower dose (200 mg/kg) showed no effect.	[174]
Cognitive and Memory Improvement (Alzheimer’s)	Mouse models with a fiber-rich diet (fructans)	Increased butyrate-producing bacteria and enhanced cognitive and spatial memory with reduced anxiety.	[175]
Alzheimer’s Disease (HDAC Regulation)	Transgenic and wild-type mouse models	Sodium butyrate supplementation improved histone acetylation, promoted learning-related genes expression, and improved memory in transgenic models.	[176,177]
Parkinson’s Disease	Mouse models	Sodium butyrate alleviated motor impairments, increased dopamine levels, and reduced neuroinflammation.	[178]
Huntington’s Disease (Histone Acetylation)	Animal models (Huntington’s disease)	Sodium butyrate restored histone acetylation and reduced apoptosis in neuronal cells, leading to increased life expectancy.	[181,182]
Mitochondrial Function and Brain Activity	Animal models exhibiting mania	Sodium butyrate replenished mitochondrial complexes and counteracted Krebs cycle inhibition.	[183,184,185]
Gut Microbiota and Inflammation	Pigs fed a diet rich in arabinoxylan	Increased beneficial butyrate-producing bacteria (e.g., *Faecalibacterium prausnitzii*), indicating a positive impact on gut health.	[186,187]
Neuroinflammation and Cognitive Impairment	Low-density lipoprotein-receptor knockout mice (HFD)	Butyrate reduced neuroinflammation, linked to decreased brain connectivity, especially in middle-aged mice.	[132]
Neuroepigenetics and Diet	Animal models with high-fiber diets	Sodium butyrate from direct supplementation showed superior neuroprotective effects compared to dietary butyrate.	[132]

Abbreviations: BTBR, Black and Tan Brachyury; HDAC, histone deacetylase; HFD, high fat diet; HPA axis, hypothalamic–pituitary–adrenal axis.

## Data Availability

No new data were created or analyzed in this study.
